# Deep Eutectic Solvents as à-la-Carte Medium for Transition-Metal-Catalyzed Organic Processes

**DOI:** 10.3390/molecules27238445

**Published:** 2022-12-02

**Authors:** Xavier Marset, Gabriela Guillena

**Affiliations:** Departamento de Química Orgánica and Instituto de Síntesis Orgánica (ISO), Facultad de Ciencias, Universidad de Alicante, Apdo. 99, 03080 Alicante, Spain

**Keywords:** deep eutectic solvent, alternative solvent, sustainable chemistry, transition metal, cross-coupling, C-H activation, multicomponent reactions, RedOx, cyclization

## Abstract

Our society is facing a tremendous challenge to become more sustainable in every sphere of life. Regarding the chemical industry, one of the most significant issues to be addressed is the use of volatile organic compounds (VOCs) as solvents because they are petrol-derived and most of them are toxic and flammable. Among the possible solutions, deep eutectic solvents (DESs) have emerged as sustainable alternatives to VOCs in organic catalyzed transformations and other fields. The advantages of these new reaction media are not only related to their more benign physical and chemical properties and, for most of them, their renewable sources but also due to the possibility of being recycled after their use, increasing the sustainability of the catalyzed process in which they are involved. However, their use as media in catalytic transformations introduces new challenges regarding the compatibility and activity of known catalysts. Therefore, designed catalysts and “*à-la-carte*” DESs systems have been developed to overcome this problem, to maximize the reaction outcomes and to allow the recyclability of the catalyst/media system. Over the last decade, the popularity of these solvents has steadily increased, with several examples of efficient metal-catalyzed organic transformations, showing the efficiency of the catalysts/DES system, compared to the related transformations carried out in VOCs. Additionally, due to the inherent properties of the DES, unknown transformations can be carried out using the appropriated catalyst/DES system. All these examples of sustainable catalytic processes are compiled in this review.

## 1. Introduction

Over the last decades, sustainability has been a key factor in modern life. Rapidly evolving society and its needs cause an increase in industrial production, and thus more environmental concerns. This trend was reflected in 2015 by the United Nations when a development plan that was focused on sustainability was published under the title “Transforming Our World: The 2030 Agenda for Sustainable Development” [1]. This trend applies to all aspects of modern society, including chemistry.

The use of solvents in the chemical industry can easily be identified as one of the major concerns when applying sustainable principles. Traditionally, volatile organic compounds (VOCs) have been employed as solvents. Most of these compounds are fossil fuel derivatives, which are not renewable, highly toxic, non-degradable, and are accumulated in the atmosphere due to their low boiling points, leaving behind a very high carbon footprint. According to recent reports, solvents constitute 80 to 90% of the non-aqueous mass employed when synthesizing an active pharmaceutical ingredient (API) [1]. Owing to these facts, finding alternative solvents is crucial to improve the sustainability of these industries.

In this sense, deep eutectic solvents (DESs) have been proposed as alternative solvents in organic chemistry. DESs are defined as systems formed from a eutectic mixture of two or more components, usually Lewis or Brønsted acids and bases that can contain a variety of anionic and/or cationic species [2]. Eutectic formation occurs through intermolecular interactions between DES constituents (with hydrogen bonds being one of the most significant ones), but there are non-covalent or ionic bonds involved. These interactions decrease the lattice energy, explaining the drop in melting point, compared with their components.

According to their composition, DESs can be divided into five main types [2,3]:

- Type I: formed by a quaternary ammonium salt and a metal chloride.
- Type II: differs from type I in having hydrated metal halides instead of non-hydrated ones.
- Type III: formed from quaternary ammonium salt and hydrogen bond donors (HBDs), e.g., alcohols, amino acids or amides.
- Type IV: formed from transition-metal salts and HBDs.
- Type V: formed exclusively by non-ionic components.

Eutectic mixtures can be synthesized by mixing their components in an appropriate ratio and heating them slightly until a melt is formed. Thus, the atom economy of the process is complete, and no by-products are formed. The easy preparation and atom economy are two of the main advantages offered by DESs, compared to the related ionic liquids. In addition to this, other advantages in their use are their negligible boiling points, the cost of their components, their renewable origin and low toxicity, and high biodegradability. These facts make them promising candidates to be used for the replacement of VOCs in organic reactions.

Not surprisingly, although early applications of DESs were related to metal extraction, electrodeposition or electropolishing were soon considered as a reaction media for organic transformations. Although, in the past 10 years, DESs proved to be useful reaction media in a myriad of organic transformations, this review will focus on transition-metal-catalyzed processes, tackling especially homogeneous catalysis. Attending to the previous classification, types I, II and IV can contain a transition metal in their composition and may act as both a solvent and catalyst, while the more environmentally friendly types III and V require the use of an external metal precursor as a reaction catalyst. In these catalytic transformations, it is essential to ensure the compatibility of the catalyst with the used DESs to provide the expected products selectively and efficiently. Due to the high polar nature of DESs, sometimes transition-metal salts can be used as a catalyst, but in most cases, the design of ligands to stabilize or modify the reactivity of the metals is required. In any case, DESs provide a unique advantage compared to VOCs, which is the possible recovery of the ligand–metal–solvent system. This allows their recyclability and enhances the sustainability of a given process. Therefore, this advantage has been highlighted in this review by including the number of reaction cycles that can be performed for each discussed example. Additionally, DESs can solubilize some gases to a greater extent, compared to VOCs and, therefore, they are excellent solvent systems to perform hydrogenations, oxidations and carbonylation reactions. In fact, through the careful choice of the appropriate DESs and catalysts, sulfonylation reactions can be performed in DESs by generating in situ SO_2_ from simple inorganic sulfur salts.

In view of greater clarity, references have been classified, according to the described transformation in the main blocks: RedOx processes, cyclization, cross-couplings, C-H functionalization, multicomponent and miscellaneous reactions. At the end of each section, a table summarizing the encountered results for each transformation has been added, providing a straightforward and clear view of the state of the art for each transformation and allowing comparisons of the achieved results.

## 2. RedOx Processes

**Figure 1:**
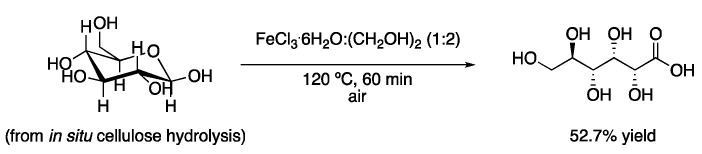
Hydrolysis and oxidation of cellulose to gluconic acid.

As commented in the introduction, types I, II and IV DESs contain metallic salts as part of their structure. Therefore, some of these eutectic mixtures can be used as solvents and catalysts at the same time. One catalytic DES system used for oxidation reactions is the mixture FeCl_3_.6H_2_O:(CH_2_OH)_2_ (2:1), which has been applied to the oxidation of cellulose to gluconic acid. The product, which could be obtained in up to 52% yield, precipitated from the reaction medium, facilitating product isolation. In addition, the DES could be reused up to five times without losing its activity with an O_2_ treatment after each cycle (Figure 1, Table 1, entry 1) [4]. Similarly, 5-hydroxymethylfurfural (HMF) has been obtained in high yields by treatment of glucose at 130 °C with the eutectic ChCl:CrCl_3_.6H_2_O in a biphasic mixture with solvents, such as EtOAc, through different dehydration processes [5].

**Figure 2:**
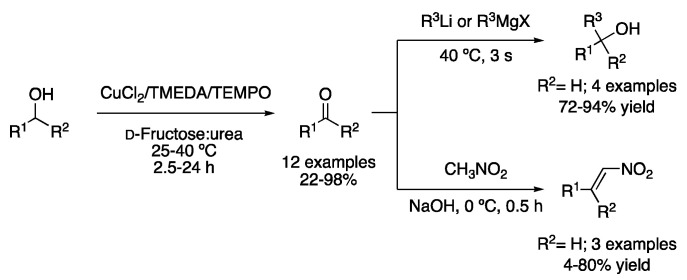
Oxidation of alcohols in DES and telescoped one-pot reactions.

Another example of a catalytic DES system was employed for the oxidation of sulfides to sulfoxides by H_2_O_2_ using catalytic amounts of the mixture ZrOCl_2_.H_2_O:urea (1:5), although MeOH was needed as a solvent. However, in the case of oxidizing aryl boronic acids to phenols, no methanol as a co-solvent was required, performing the reaction with only DESs and 30% aqueous H_2_O_2_ as the solvent. The DES dissolved in water could be concentrated and reused up to three times, observing a slight decrease in the reaction yield [6]. Much more recently, the catalytic system based on CuCl_2_, TMEDA and TEMPO was employed in water and in the mixture d-fructose:urea (3:2) to perform the selective aerobic oxidation of alcohols. In addition, telescoped one-pot-hybrid reactions are feasible, in such a way that either an addition of Grignard reagents or a Henry reaction can be performed on the in situ generated aldehyde (Figure 2, Table 1, entry 2) [7].

Other oxidations are based on heterogeneous catalysts, such as metal-organic frameworks. These catalysts have been used in combination with DESs to perform reactions, such as the synthesis of HMF from carbohydrates [8] or the epoxidation of styrene [9].

Hydrogenation and hydroformylation reactions have been more extensively studied. One of the earliest examples was performed in a mixture of DMU and a ß-cyclodextrin derivative as a solvent, using Rh(acac)(CO)_2_ in combination with the ligand triphenylphosphine-3,3′,3′′-trisulfonic acid trisodium salt. This ionic phosphine derivative enhanced the compatibility of the catalysts with the polar solvent. Therefore, 1-decene was converted at 90 °C, 1 h into the corresponding aldehyde using 50 bar of a mixture of CO/H_2_, affording a ratio of the linear to the branched product of 2.3:1 [10]. Further studies by the same group on this topic using different solvent mixtures determined that the conversion was highly dependent on the solubility of the substrate and inversely dependent on the solvent viscosity [11]. The hydroformylation of 1-decene was also studied in different choline chloride-based eutectic mixtures. After screening different conditions, including several eutectic mixtures and ligands, the best results were obtained with Rh(acac)(CO)_2_ using BiPhePhos as a ligand in ChCl:urea (1:2) as reaction media. In this way, the yield of aldehydes was 41%, showing high selectivity to the linear aldehyde over the branched one (97:3, Figure 3, Table 1, entry 3) [12].

**Figure 4:**
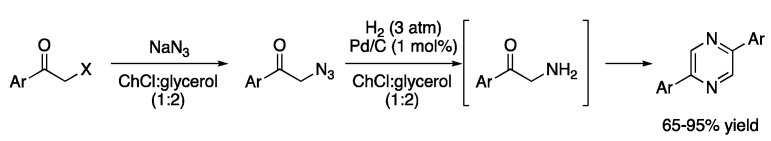
Hydrogenation of phenacyl azides.

Palladium-catalyzed hydrogenations have also been investigated in DESs media, although given the nature of these reactions, most of the systems are based on heterogeneous catalysts. One of the earliest examples is the Pd/C-catalyzed hydrogenation of phenacyl azides in ChCl:glycerol (1:2), providing intermediates, which yielded symmetrical 2,5-diarylpyrazines. In addition, the nucleophilic substitution of α-halo ketones with sodium azide can be performed in a one-pot manner using the eutectic mixture as a solvent (Figure 4, Table 1, entry 4) [13].

**Figure 5:**
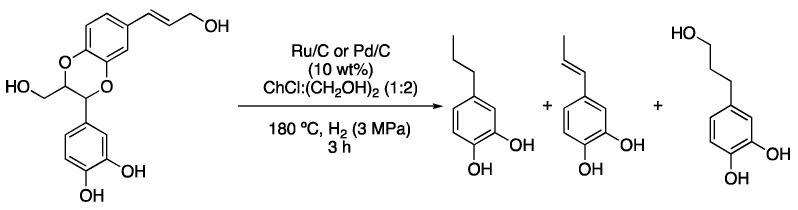
Hydrogenolysis of benzodioxane derivative.

The use of a mixture of choline tosylalaninate with glycerol was reported for performing Pd-NPs-catalyzed hydrogenations of 4-phenylbut-3-en-2-one and 4-phenylbutan-2-one. An interesting effect must be pointed out. This is the fact that the use of high pressures of mixtures of CO_2_ and H_2_ improved the results over the use of pure H_2_ atmospheres. It was rationalized that high pressures of CO_2_ decreased the viscosity of the DESs while improving the diffusion coefficient of H_2_ and reactants to almost two times their initial values, thus enhancing the catalytic activity [14]. The depolymerization of lignin is another pertinent topic in current sustainable chemistry. A linear homopolymer can be found in castor seed coats and a model benzodioxane compound present in this polymer was subjected to hydrogenolysis using Pd/C or Ru/C at 180 °C for 3 h under 3 MPa H_2_ atmosphere and ChCl:(CH_2_OH)_2_ (1:2) as a solvent (Figure 5, Table 1, entry 5) [15].

**Figure 6:**
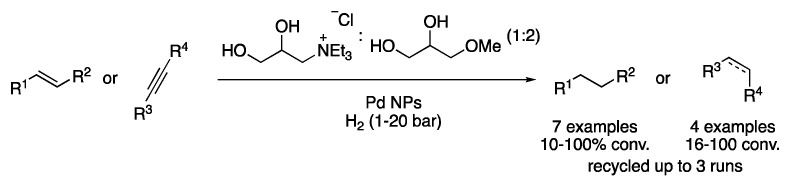
Pd-NPs-catalyzed hydrogenation of unsaturated compounds.

Pd NPs have also been synthetized directly on DES based on bio-ammonium salts and glycerol methyl ethers. The corresponding solutions were applied to the hydrogenation of alkenes, alkynes and carbonyl compounds (Figure 6) [16].

Other hydrogenation processes involve the use of DESs to prepare supported metallic catalysts, although the hydrogenation reactions are performed in other solvents, as is the case of Pd supported on pyrolyzed DES [17], Pd-DES@SiO_2_ [18], Pd immobilized on a composite of halloysite and DES [19] or gold nanoparticles dispersed in ChCl:urea (1:2) [20].

**Figure 7:**
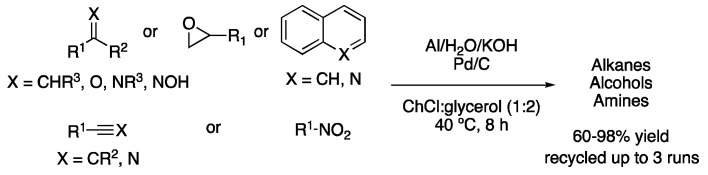
Hydrogenation using in situ generated H_2_ from Al and H_2_O.

All these DES-mediated procedures try to reduce the environmental impact of chemical transformations by using sustainable solvents. Hydrogenations, however, fail to follow another principle of green chemistry, namely, safety, since hydrogenations require high hydrogen pressures. To avoid that, a safer method was developed by the in situ generation of hydrogen from aluminum powder and water in ChCl:glycerol (1:2) as a solvent. Thus, the generated hydrogen was employed to reduce a wide variety of functional groups, such as aldehydes, epoxides, nitro-compounds, imines, nitriles, alkenes or alkynes in good yields at only 40 °C in 8 h reaction time (Figure 7, Table 1, entry 6) [21].

**Figure 8:**
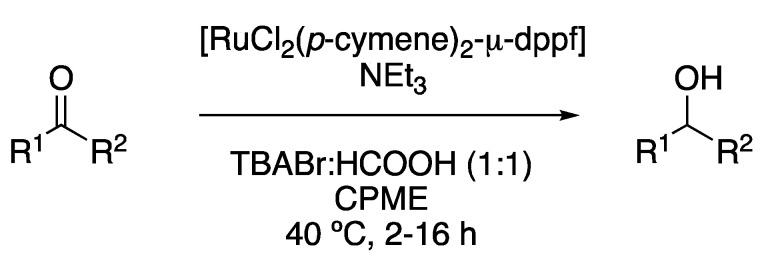
Ru-catalyzed transfer hydrogenation.

To conclude, although most hydrogenations are performed using a palladium catalyst, a Ru(II)-transfer hydrogenation of carbonyl compounds was described in the biphasic mixture of TBABr:HCOOH (1:1) and cyclopentyl methyl ether (CPME). In this case, the eutectic mixture acts as a reaction medium, as well as a hydrogen source, with the complex [RuCl(p-cymene)Cl_2_]_2_ in combination with the ligand 1,1′-Ferrocenediyl-bis(diphenylphosphine) (dppf) being the catalyst. Reactions were performed between 40 and 80 °C (depending on the substrate reactivity) using triethylamine as a base (Figure 8, Table 1, entry 7) [22].

**Table 1:** Selected examples of RedOx processes catalyzed by transition metals in DESs.

Entry	Reaction	DES	Conditions	Product	Ref.
1	Hydrolysis and oxidation	FeCl_3·_6H_2_O:(CH_2_OH)_2_ (1:2)	120 °C	Gluconic acid	[4]
2	Alcohol Oxidation	d-fructose:urea (3:2)	25–40 °C	Ketones/Aldehydes	[7]
3	Hydroformylation	DMU: RAME-ß-CD	CO/H_2_ (50 bar)	Aldehydes	[12]
4	Hydrogenations	ChCl:glycerol (1:2)	Pd/C, H_2_	Diarylpyrazines	[13]
5	Hydrogenolysis	ChCl:(CH_2_OH)_2_ (1:2)	Ru/C or Pd/C, H_2_	Resorcinol derivatives	[15]
6	Hydrogenation	ChCl:glycerol (1:2)	Al, KOH, H_2_O	Alkanes/alcohols/amines	[21]
7	Transfer hydrogenation	TBAB:HCOOH (1:1)	[Ru], NEt_3_, CPME	Secondary alcohols	[22]

## 3. Cyclization Reactions

Heterocyclic compounds are broadly present in nature, as well as in biologically active compounds used in the pharmaceutical industry. Therefore, loads of synthetic methods have been developed, with several strategies being carried out in DESs as reaction media. Two main strategies to generate new rings will be discussed, i.e., cycloadditions and condensation reactions.

### 3.1. Cycloadditions

**Figure 9:**
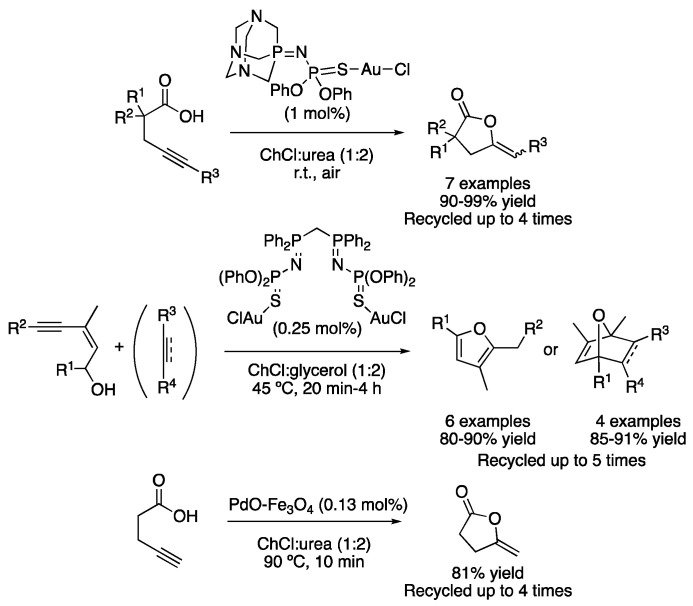
Cycloisomerization reactions catalyzed by Au and Pd.

One of the first examples of cycloadditions in DESs is based on the cycloisomerization of γ-alkynoic acids in ChCl:urea (1:2) using a gold-based catalyst. The reaction was performed under air atmosphere at room temperature and completed in 0.25–3.5 h, with the catalyst being reusable in up to four cycles without losing its catalytic activity (Table 2, entry 1) [23]. Shortly after, the same group reported the cycloisomerization of (Z)-enynols into furans. In that case, a bis(iminophosphorane)-Au(I) complex was used in the mixture ChCl:glycerol (1:2) as a reaction medium in the absence of any other co-catalyst. In addition, the in situ generated furan derivative could be further transformed via a Diels–Alder reaction in a one-pot manner (Table 2, entry 2) [24]. Another iminophosphorane-Au complex was also described to catalyze the cycloisomerization of various alkynyl amides into alkylidene lactams using water or ChCl:urea (1:2) as solvents in good-to-excellent yields [25]. Moreover, the cycloisomerization of γ-alkynoic acids was described using a recyclable palladium supported on a magnetite catalyst. Although water was found to be an excellent solvent for this transformation, the reaction also proceeded with good yields using the mixture ChCl:urea (1:2) at 90 °C in 10 min reaction time (Figure 9) [26].

**Figure 10:**
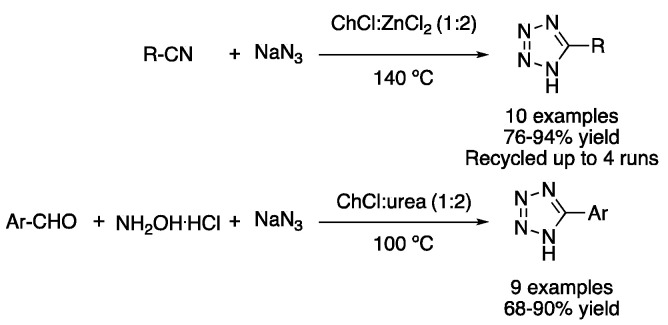
Tetrazole syntheses through [3 + 2] cycloadditions.

Other cycloadditions take advantage of the properties of type I DESs, using them as solvents and catalysts. In this sense, the mixture ChCl:ZnCl_2_ (1:2) was employed for promoting the [2 + 3] cycloaddition of nitriles and sodium azide. The reaction, performed at 140 °C for 0.5–12 h, afforded excellent yields of 5-substituted 1H-tetrazoles using aromatic or benzylic nitriles as starting materials [27]. However, the more sustainable mixture ChCl:urea (1:2) can also be used to carry out this transformation using Cu(OAc)_2_ as catalyst. Thus, the one-pot reaction of aromatic aldehydes, hydroxylamine hydrochloride and sodium azide was performed at 100 °C for 12 h (Figure 10, Table 2, entry 3) [28].

**Figure 11:**
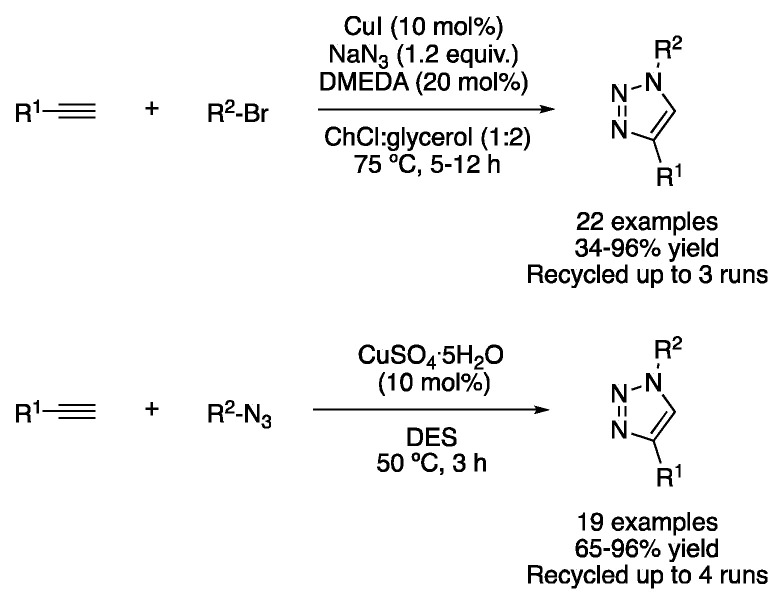
Syntheses of triazole derivatives.

Similarly, the synthesis of triazoles via copper-catalyzed azide–alkyne cycloaddition (CuAAc) has been described in DESs media. Namely, ChCl:glycerol (1:2) was employed as a solvent to perform the copper-catalyzed azidation of aryl bromides, followed by the cycloaddition to an alkyne to yield the corresponding 1,4-disubstituted triazole derivatives. In addition, the catalytic system could be reused several times, although results were improved when fresh ligand (*N,N*′-dimethylethylendiamine) was added after the third cycle [29]. NADES have also been shown to promote this transformation, acting both as solvents and in stabilizing catalytic copper intermediates, as well as by interacting with the alkyne through the H bond, as demonstrated by DFT calculations. Thus, the mixtures ascorbic acid:choline chloride (1:2) and glycolic acid:trimethylglycine (2:1) were effectively used as solvents and avoided the need of using external bases or reducing agents, with the solvent being recyclable up to three times without loss of activity (Table 2, entry 4) [30]. In addition, a heterogeneous catalyst based on copper iodide supported on 3-thionicotinyl-urea-modified magnetic nanoparticles was also employed to perform this transformation using ChCl:PEG (1:4) as a solvent (Figure 11) [31].

### 3.2. Cyclizations through Condensation

**Figure 12:**
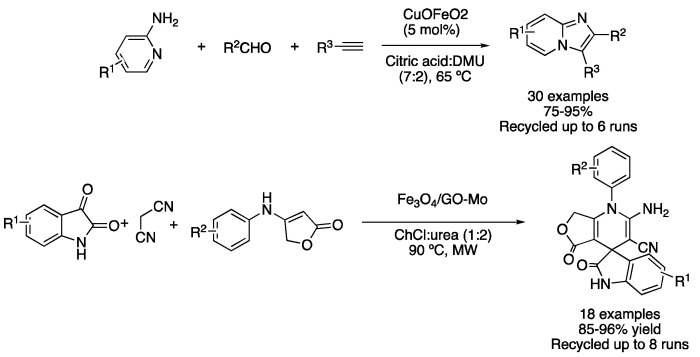
Heterocycle synthesis using heterogeneous catalysts in DESs.

Condensation reactions are a second approach to perform cyclization reactions. Early reports of condensation-based cyclizations in DES media were reported using heterogeneous catalysts. For instance, the mixture citric acid:DMU (7:2) was used as a solvent for performing the synthesis of [1,2-a]pyridines using 5 mol% of recyclable CuFeO_2_ to catalyze the reaction between 2-aminopyridine derivatives, aldehydes and alkynes. The reaction did not proceed in traditional solvents under these conditions. However, the use of an acid as a component of the eutectic mixture was crucial to obtaining good yields, proving that DES played a double role, both as a solvent and also as an acidic catalyst (Table 2, entry 5) [32]. These compounds have also been prepared in TBAB:glycerol (1:2) using copper iodide supported on magnetic nanoparticles [33]. Another example of a heterogeneous catalyst for performing cyclization reactions in DESs is based on molybdenum supported on graphene oxide/Fe_3_O_4_, which was employed for the synthesis of spiro-oxindole dihydropyridines in ChCl:urea (1:2). These reactions were carried out by the combination of isatins, malononitrile and anilinolactones at 90 °C under microwave irradiation. The catalytic system could be reused up to eight times, showing almost negligible leaching for Fe and Mo in the ChCl:urea (1:2) phase (Figure 12, Table 2, entry 6) [34]. Similarly, 2,3-dihydroquinazolin-4-(1H)-ones and spiroquinazolin-4(3H)-ones derivatives were prepared with Fe_3_O_4_@SiO_2_@TiO_2_-OSO_3_H magnetic nanoparticles as a catalyst in the mixture ChCl:urea (1:2), by reaction of isatonic anhydride, anilines (or ammonium chloride) and cyclic aliphatic ketones or benzaldehyde derivatives [35]. The same eutectic mixture was also employed for the preparation of 7-aryl-benzo[h]tetrazolo[5,1-b]quinazoline-5,6-dione, employing a magnetic polymeric nanostructure, with DES-catalyst being recyclable up to six times [36]. Finally, a DES-coated metal-organic framework was employed for the multicomponent synthesis of 2-amino-4H-chromenes, although, in this case, reactions were carried out in a solvent-free fashion [37].

**Figure 13:**
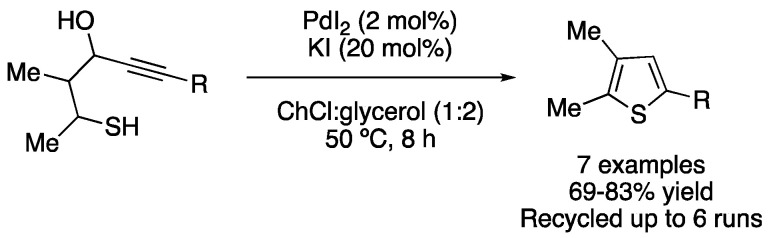
Synthesis of substituted thiophenes by PdI_2_/KI-catalyzed heterocyclization.

The S-heterocyclodehydration of 1-mercapto-3-yn-2-ols catalyzed by PdI_2_ was reported in ChCl:glycerol (1:2) at 50 °C, affording seven examples of thiophene derivatives with good yields. The catalytic system was recycled up to six times without any loss of activity (Figure 13; Table 2, entry 7). Moreover, the starting material of this reaction could also be prepared in eutectic solvent as reaction media [38].

**Figure 14:**
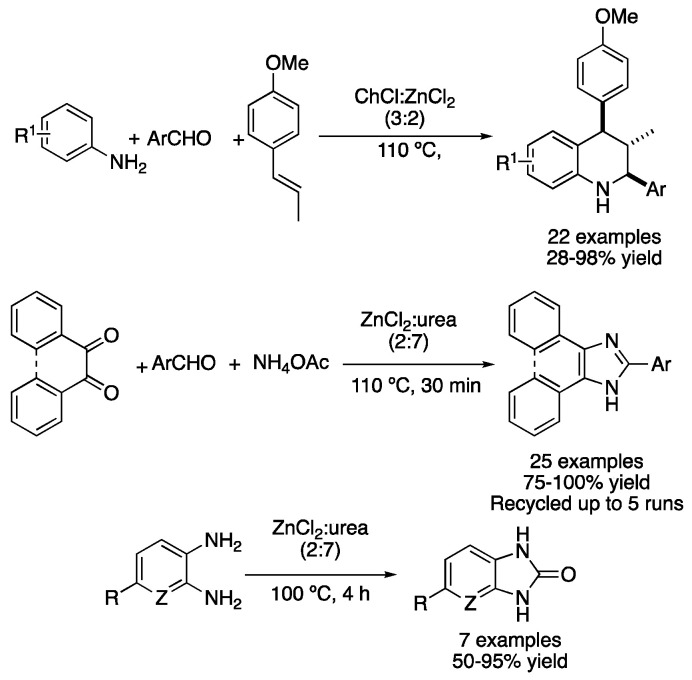
Condensation-based cyclization reactions using Zn-based type I and IV DESs.

Due to the nature of most condensation reactions, the presence of a Lewis acid catalyst is beneficial for the reaction to take place in good yields. Thus, type II DESs based on hydrated metallic salts and quaternary ammonium salts can be applied not only as solvents but also as catalysts to perform these transformations. In this sense, the mixture ChCl:ZnCl_2_ (3:2) was used for synthesizing tetrahydroquinolines from anilines, aromatic aldehydes and trans-anethole, a naturally occurring essential oil. Firstly, condensation takes place to form an imine derivative, followed by an aza-Diels–Alder reaction to afford the desired heterocycle. Moderate-to-excellent yields were obtained with high diastereoselectivity and wide substrate scope (Table 2, entry 8) [39]. The same eutectic mixture was also reported as a catalyst for the synthesis of 4-aryldeneisoxazol-5(4H)-ones, although the reaction was performed in water as a solvent [40]. In addition, the type-IV mixture ZnCl_2_:urea (2:7) has also been employed as a catalytic solvent for performing reactions, such as the synthesis of 2,4,5-triaryl-1H-imidazoles and 2-aryl-1H-phenantro[9,10-d]imidazoles. Those compounds were prepared by the reaction between dicarbonyl compounds, ammonium acetate and aromatic aldehydes. Up to 25 examples were reported with excellent yields, with the DES being recovered by dissolution in water, filtration and lyophilization. This allowed the DES to be reused up to four times with only a slight decrease in the catalytic activity (Table 2, entry 9) [41]. Another heterocyclic synthesis in ZnCl_2_:urea (2:7) is the synthesis of 2-benzimidazolones and 2-imidazolones, which relies on a triple role for the DES, i.e., the eutectic mixture acts as a solvent, as a Lewis acid catalyst and as a reagent (urea). Therefore, arene-1,2-diamine derivatives were heated at 100 °C for 4 h in the aforementioned DES, affording seven examples of the corresponding 2-benzimidazolone derivatives in moderate-to-excellent yields. If benzoin derivates were employed instead of the diamine compounds, the corresponding 2-imidazolone products could be obtained in good yields, employing a method that avoids the generation of gas ammonia evolution (Figure 14, Table 2, entry 10) [42].

**Figure 15:**
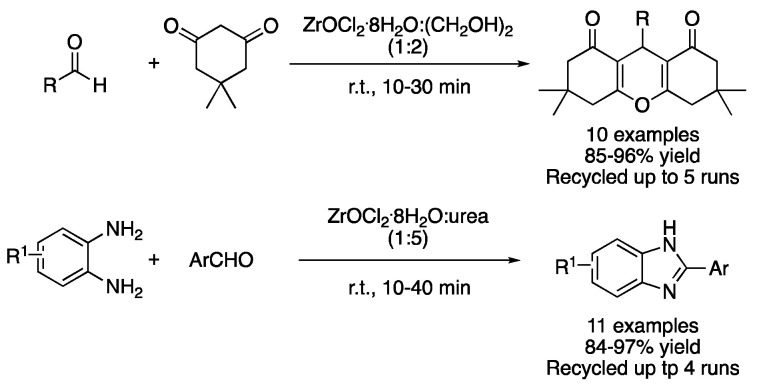
Cyclizations using Zr-based type IV DESs.

Apart from ZnCl_2_-based DES, the use of ZrOCl_2_.8H_2_O in combination with different hydrogen bond donors (HBD) has also been explored. One example is the synthesis of 1,8-dioxooctahydroxanthenes from dimedone and aromatic aldehydes. This reaction was explored using urea, ethylene glycol and glycerol as HBD. The best results were obtained with the mixture ZrOCl_2_.8H_2_O:(CH_2_OH)_2_ (1:2), which was explained by the ability of ethylene glycol to activate the aldehyde through hydrogen bonding and the lower viscosity of the mixture, compared to the glycerol one, which favors the free motion of reactants. Furthermore, DES could be recovered and reused up to five times without a noticeable loss in its catalytic activity (Figure 15, Table 2, entry 11) [43]. However, the synthesis of benzimidazole derivatives was far more efficient in the mixture ZrOCl_2_.8H_2_O:urea (1:5), which was achieved by the reaction of arene-1,2-diamine with aromatic aldehydes. The main reasons behind the good results obtained with this DES are the higher acidity and lower viscosity of this mixture compared with their ethylene glycol or glycerol HBD analogues (Table 2, entry 12) [44].

**Table 2:** Selected examples of cyclization processes catalyzed by transition metals in DESs.

Entry	Reaction	DES	Conditions	Product	Ref.
1	Cycloisomerization of alkynoic acids	ChCl:urea (1:2)	[Au]	Enol-lactones	[23,26]
2	Cycloisomerization of Z-enynols	ChCl:glycerol (1:2)	[Au]	Furans	[24]
3	3+2 Cycloaddition	ChCl:ZnCl_2_ (1:2)	100–140 °C	1*H*-Tetrazoles	[27,28]
4	3+2 Cycloaddition	ChCl:glycerol (1:2)	[Cu], 50–75 °C	Triazoles	[29,30]
5	A^3^ coupling	Citric acid:DMU (7:2)	CuFeO_2_	imidazo[1,2*a*]pyridines	[32,33]
6	Condensation	ChCl:urea (1:2)	[Fe/Mo], MW	spirooxyndoles	[34]
7	S-heterocyclization	ChCl:glycerol (1:2)	PdI_2_/KI	Thiophene derivatives	[38]
8	Condensation + D.A.	ChCl:ZnCl_2_ (3:2)	110 °C	Tetrahydroisoquinolines	[39]
9	Condensation	ZnCl_2_:urea (2:7)	110 °C	phenantro[9,10*a*]imidazoles	[41]
10	Condensation	ZnCl_2_:urea (2:7)	100 °C	2-benzimidazolones	[42]
11	Condensation	ZrOCl_2_:(CH_2_OH)_2_ (1:2)	r.t.	dioxooctahydroxanthenes	[43]
12	Condensation	ZrOCl_2_:urea (1:5)	r.t.	benzimidazoles	[44]

## 4. Cross-Coupling Reactions

**Figure 16:**
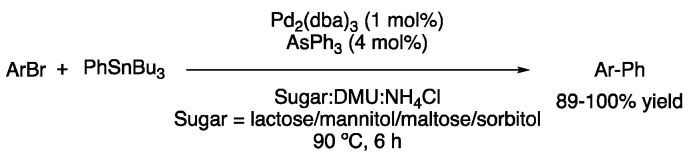
First example of a cross-coupling reaction in DESs.

One of the first reports of metal-catalyzed processes in DESs was the biaryl synthesis through Stille reaction in mixtures of sugars, dimethylurea and ammonium chloride. Alkylation and arylations were performed with aryl bromides as coupling partners, obtaining excellent yields. It is worth mentioning that this reaction was performed under palladium catalysis using triphenyl arsine as the ligand, while using the more common (2-biphenyl)dicyclohexylphosphine resulted in a lower yield. Melt and catalyst could be reused three times, showing a slow conversion decrease after each cycle (Figure 16) [45].

**Figure 17:**
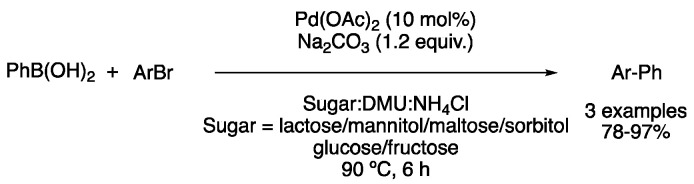
First Suzuki reaction in DESs.

When it comes to transition-metal-catalyzed cross-coupling reactions, there is one that stands out, the Suzuki–Miyaura coupling. In fact, the first Suzuki reaction performed in DESs was already reported in 2006 by the König group. At that time, three examples of biaryl products were obtained from the reaction between phenylboronic acid and different aryl bromides, using 10 mol% of Pd(OAc)_2_ and sodium carbonate as a base in mixtures of sugars–urea and chloride salts at 90 °C with yields ranging from 78 to 97% (Figure 17) [46].

**Figure 18:**
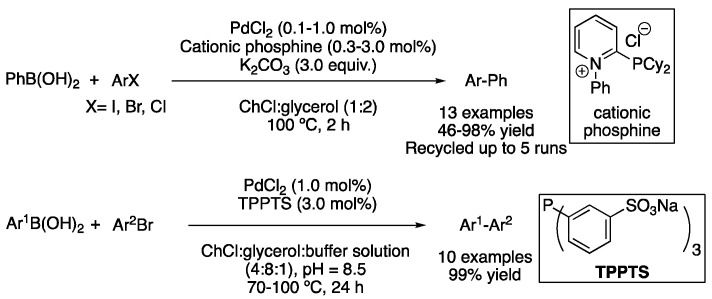
Phosphine ligands in cross-coupling reactions in DESs.

Eight years after the first report, the use of ß-cyclodextrin-capped Pd^0^ nanoparticles (NPs) was described in the mixture ß-CD/*N*-methyl urea. In this case, K_2_CO_3_ was used as a base and only 0.05 mol% catalyst loading was required when the reaction was carried out with aryl iodides and bromides, while 0.5 mol% and longer reaction times were employed in the case of aryl chlorides. Anyhow, good-to-excellent yields were obtained for the coupling of phenyl boronic acid with different aryl halides [47]. The ligand effect for achieving Suzuki–Miyaura couplings in DESs was first studied in 2017 by Ramón’s group. It was found that traditional ligands were not as efficient in DESs as they were in common organic solvents. Therefore, pyridiniophosphine ligands were synthesized, which were found to perform better than their non-ionic analogues. After this small structural modification, these ligands in combination with 1 mol% of PdCl_2_ were used to perform the Suzuki–Miyaura coupling between PhB(OH)_2_ and aryl bromides and in just 2 h using ChCl:glycerol (1:2) as a solvent at 100 °C. In the case of using more reactive aryl iodides instead of bromides, only 0.1 mol% catalyst loading was required. In addition, catalysts and solvents could be recycled up to five times without losing their catalytic activity, taking advantage of the unique properties of DESs. The same catalytic system was also efficiently employed on the Sonogashira and Heck reactions [48]. Another ionic phosphine ligand, namely the commercially available triphenylphosphine-3,3′,3′′-trisulfonic acid trisodium salt (TPPTS) was also employed in combination with PdCl_2_ in ChCl:glycerol (1:2), although in this case, an aqueous buffer (pH 8.5) was also added. Several biaryls containing ketone substituents were synthesized with excellent yields, and those products were used to perform the enzyme-catalyzed enantioselective reduction to benzylic alcohols [49]. A similar strategy was later applied for the enzymatic transamination of biaryl ketones obtained by Suzuki coupling in DES (Figure 18) [50].

**Figure 19:**
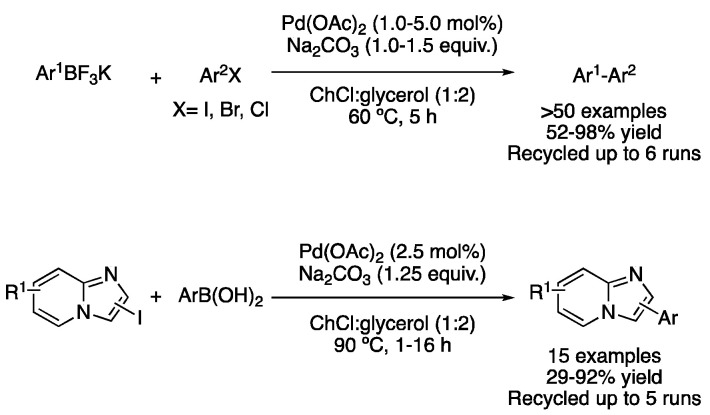
Ligand-free Suzuki couplings.

ChCl:glycerol (1:2) was also employed as a solvent for the ligand-free coupling of aryl trifluoroborates with aryl iodides, bromides and chlorides with excellent yields using Pd(OAc)_2_ (1–5 mol%) and Na_2_CO_3_ (1.0–1.5 equiv.) [51]. Not only biphenyls were synthesized in this report but also the same strategy was employed for the synthesis of terphenyls with moderate-to-excellent yields. It is worth mentioning that the catalytic system could be recycled up to four times when 1 mol% Pd loading was employed, or up to five times when using the initial 5 mol% catalyst loading. An interesting application of Suzuki–Miyaura coupling in DESs was reported by Delaye et al. performing the reaction between aryl boronic acids and imidazole-fused heterocycle iodides. Good results were obtained with ChCl:glycerol (1:2) as the solvent, with the catalytic system being recyclable up to five times (Figure 19) [52].

**Figure 20:**
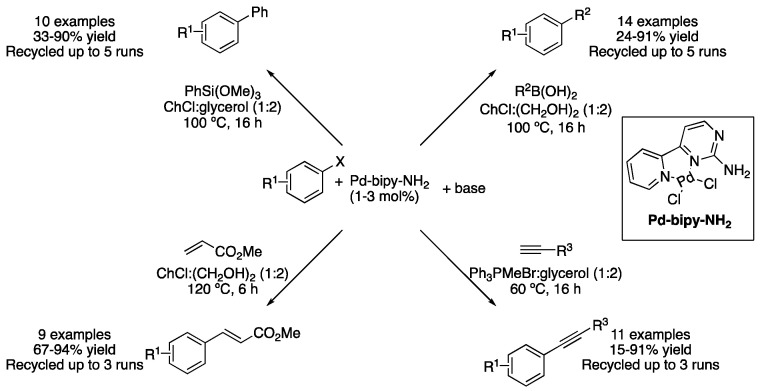
Pd-bipy-NH_2_ based cross-coupling transformations.

A novel bipyridine–palladium complex was reported to catalyze several cross-coupling reactions. Due to the hydrogen bond formation capability of the ligand with DESs components, the compatibility of the air-stable pre-catalyst was improved, being effective at performing Hiyama, Suzuki–Miyaura, Heck–Mizoroki and Sonogashira cross-coupling reactions. The most efficient solvents for the aforementioned reactions were found to be ChCl:glycerol (1:2) for Hiyama, ChCl:(CH_2_OH)_2_ (1:2) for Suzuki and Heck and Ph_3_PMeBr:glycerol (1:2) for Sonogashira couplings. An amount of 1–3 mol% of pre-catalyst were used. The catalytic system could be recycled up to five times for the Hiyama and Suzuki reactions and up to three times for the case of Heck and Sonogashira couplings. TEM and XPS analyses of the residue form, after the reaction completion, indicated that Pd NPs were formed during the reaction course. A mercury test proved that those were the active catalytic species. Therefore, the bipyridine–palladium complex acted as a pre-catalyst and helped to stabilize the in situ generated NPS (Figure 20) [53].

**Figure 21:**
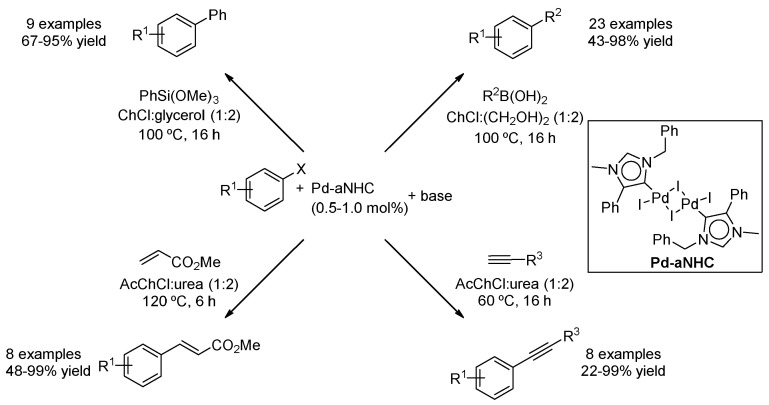
Pd-abnormal *N*-heterocyclic carbene catalyzed cross-coupling reactions.

Similar behaviour was observed with a palladium mesoionic carbene pre-catalyst. In this case, the mixture AcChCl:urea (1:2) was found to be more effective for the Sonogashira and Heck couplings, while ChCl:glycerol (1:2) and ChCl:(CH_2_OH)_2_ (1:2) were still the preferred choice for Hiyama and Suzuki couplings, respectively. Regarding this latest reaction, it is worth mentioning that only 0.5 mol% catalyst loading was employed, and good-to-excellent yields were obtained using aryl bromides and even chlorides. In addition, biaryl products containing ketone functionalities were used to perform organometallic additions in DESs (Figure 21) [54].

**Figure 22:**
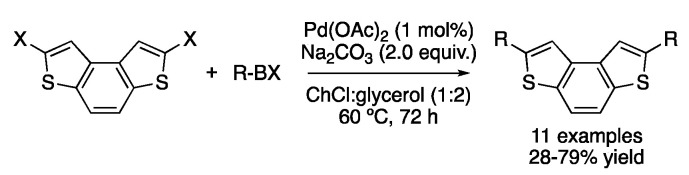
Application of Suzuki coupling to benzodithiophene derivatives.

Another interesting application of Suzuki–Miyaura coupling in DESs proposed the regioselective ortho-lithiation of aryl amides followed by iodination to obtain the corresponding aryl iodide, which is directly used to perform a Suzuki–Miyaura coupling catalyzed by Pd(OAc)_2_ in ChCl:glycerol (1:2)/CPME [55]. The symmetric arylation and alkenylation of benzodithiophenes were also described in the mixture ChCl:glycerol (1:2) as the solvent, yielding products with potential electrochromic properties (Figure 22) [56].

**Figure 23:**
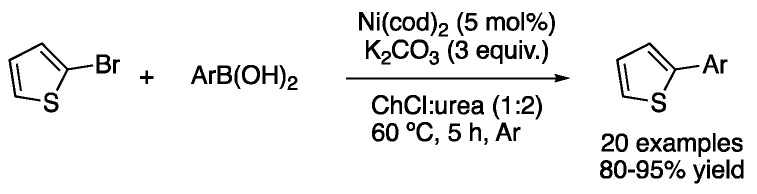
Ni-catalyzed Suzuki-Miyaura coupling.

In addition, there is one report in which the Suzuki–Miyaura coupling in DESs is performed with a Ni catalyst instead of a Pd one. In this case, 5 mol% of Ni(cod)_2_ is employed to carry out the reaction between 2-bromothiophene and several aryl boronic acids in ChCl:urea (1:2) as the solvent, using potassium carbonate as a base for 5 h at 60 °C. Good-to-excellent yields were obtained for the 20 reported examples (Figure 23) [57].

**Figure 24:**
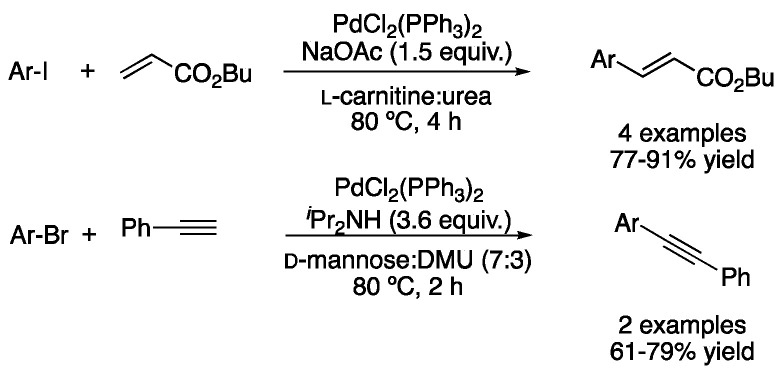
Early examples of Heck and Sonogashira couplings in DESs.

Besides the Suzuki–Miyaura coupling for the synthesis of biaryls, other well-studied palladium-catalyzed C-C cross-coupling reactions include Heck and Sonogashira reactions. One of the earliest reports was published by König in 2009. An example of Heck reaction between 1-bromo-4-iodobenzene and *n*-butyl acrylate was reported using sodium acetate as a base and palladium acetate as the catalyst at 80 °C in the mixture *L*-carnitine:urea in 75% yield. In the same study, the Sonogashira coupling between phenylacetylene and two aryl bromides was reported in the mixture d-mannose:DMU at 80 °C. In that case, PdCl_2_(PPh_3_)_2_ was employed as a catalyst and *^i^*PrNH_2_ as a base. Reactions were carried out for 2 h at 80 °C, affording the coupling products in 61–79% yield (Figure 24) [58].

Some applications of the Sonogashira coupling in DESs include the synthesis of hydrogen-substituted graphynes through the reaction of 1,3,5-tribromobenzene and 1,3,5-triethynylbenzene catalyzed by Pd(PPh_3_)_4_/CuI with Et_3_N as a base. ChCl:(CH_2_OH)_2_ (1:3), ChCl:phenol (1:2) and ChCl:urea (1:2) were used as solvents, observing different morphologies on the obtained graphynes depending on the solvent employed [59]. Furthermore, the cyclization of 6-bromo-2-chloro quinoline-3-carbaldehyde with 2-amino phenol was reported in the mixture K_2_CO_3_:(CH_2_OH)_2_ (1:10), affording an intermediate, which was subsequently employed to perform Pd-catalyzed Suzuki or Sonogashira reactions in a one-pot manner [60]. The iodocyclization of 2-methylthiophenylacetylenes to 3-iodobenzothiophenes was also described, followed by Sonogashira or Suzuki couplings in the mixtures ChCl:glycerol (1:2). A total of 17 examples of coupling products were reported with good-to-excellent yields, with the catalysts and DESs being recyclable up to five times without any loss in the catalytic activity [61]. Regarding the Heck coupling, González-Sabín and García-Álvarez described the one-pot chemoenzymatic decarboxylation of *p*-hydroxicynnamic acid, followed by a Pd(PPh_3_)_4_-catalyzed Heck reaction with PhI in a mixture of ChCl:glycerol:water (1:2:1, Figure 25) [62].

Although the main goal of this review is to describe homogeneous catalysts in DESs, it is worth mentioning that some authors have employed palladium NPs using diverse supports, such as ß-cyclodextrin@graphene [63], DNA-Fe_3_O_4_ [64], magnetically retrievable phosphine-functionalized cellulose (Fe_3_O_4_@PFC-Pd(0) [65], cellulose-modified Fe_3_O_4_/GO [66,67], to perform reactions, such as Suzuki or Hiyama couplings. Other reactions performed in DESs based on heterogeneous catalysts include the Sonogashira coupling using Pd/C in ChCl:glycerol (1:2) [68], Pd-cellulose-GO@Fe_3_O_3_ to perform Sonogashira and Heck couplings in dimethylammonium chloride:glycerol (1:2) as a solvent [69], Pd NPs@poly-*N*-vinylpirrolidone catalyzing Heck reactions in glycerol-derived DESs [70] and a Pd-MOF as catalyst of Heck cross-coupling in the ternary mixture ChCl:(CH_2_OH)_2_:DABCO (1:2:1) [71]. Ullmann homocoupling between aryl iodides has been described with Pd NPs@pentaamide-f-multi-wall carbon nanotubes in ChCl:glycerol (1:2) [72]. More recently, the use of Pd/C and Ca(OH)_2_ has been reported to efficiently catalyze the homocoupling of electron-deficient (hetero)aryl chlorides in the same solvent [73].

**Figure 26:**
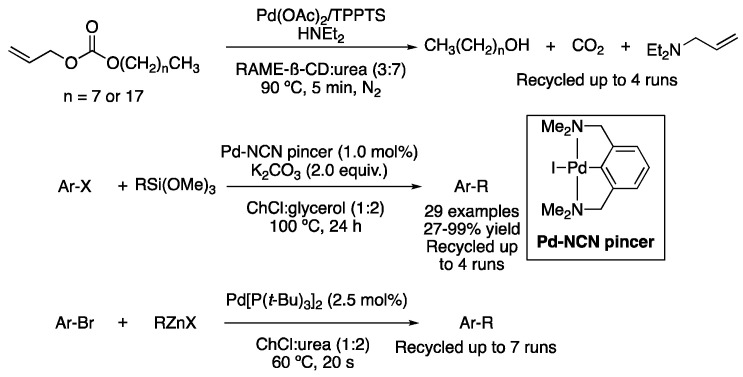
Tsuji-Trost, Hiyama and Negishi couplings in DESs.

In addition, other Pd-catalyzed cross-coupling reactions performed in DES include Tsuji–Trost, Hiyama or Negishi couplings. In 2014, Jérôme et al. described two examples of cleavage of allyl alkyl carbonates with diethylamine at 90 °C using Pd/TPPTS catalytic system in a mixture of DMU and a ß-cyclodextrin derivative as a solvent. Quantitative yields were obtained in 5 min, with the catalyst and solvent being reused for up to four cycles without loss of activity [10]. Regarding the Hiyama coupling, a palladium NCN–pincer complex was used as a catalyst in the reaction between aryl-, vinyl- and allyl- trimethoxysilanes with aryl iodides and bromides using potassium carbonate as a base. Those reactions were performed in ChCl:glycerol (1:2) as a solvent, as well as in neat glycerol for comparison. In general, slightly better results were obtained in neat glycerol. However, the use of DES improved the recyclability process, with the solvent and catalyst being reused for up to four cycles without losing their catalytic activity [74]. More recently, the reaction between organozinc compounds and (hetero)aryl bromides have been reported in ChCl:urea (1:2) using Pd[P(*t*-Bu)_3_]_2_ as a catalyst at 60 °C. The reaction takes place in just 20 s, obtaining yields ranging from 35% to 95%. Interestingly, the reaction in water with 1 equiv. of NaCl afforded even better results in most cases (Figure 26) [75].

**Figure 27:**
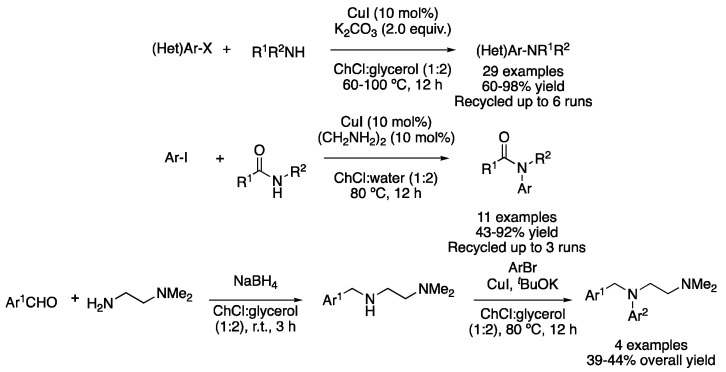
C-N cross-coupling reactions.

C-N cross-coupling reactions have also been explored in DESs as reaction media. CuI-catalyzed Ullmann amine synthesis was reported in ChCl:glycerol (1:2) using K_2_CO_3_ as a base in the case of aliphatic primary and secondary amines and *t*BuOK as a base for aromatic amines at temperatures between 60–100 °C. Good-to-excellent yields were obtained for (hetero)aryl iodides and bromides, with the catalyst, base and solvent being recyclable up to six times [76]. Similarly, Goldberg-type C-N coupling between aryl iodides and amides was reported using CuI, ethylene diamine as ligand, KOH as a base and ChCl:H_2_O (1:2) as a solvent at 80 °C for 12 h, obtaining moderate-to-excellent yields (Figure 27) [77]. Very recently, a two-step approach for the synthesis of the antihistamine drug Thenfadil and some analogues has been developed in the mixture ChCl:glycerol (1:2). The first step is based on a reductive amination between an aldehyde and a primary amine using NaBH_4_, and then an Ullman coupling takes places on the generated secondary amine to afford the desired compounds. It is worth noting that no chromatographic steps were required to purify the products and that the reaction was scaled up to 50 g [78] (Figure 27). Other C-N coupling transformations based on supported heterogeneous catalysts include: Cu-NPs-carboxamide-*f*-GO@Fe_3_O_4_ [79], Cu(I)-creatine@Fe_3_O_4_ [80], Cu(I)-Si(CH_2_)_3_*N*-acyclovir-SiO_2_@Fe_3_O_4_ [81] and Pd(II)-vitamin B6@Fe_3_O_4_ [82].

**Figure 28:**
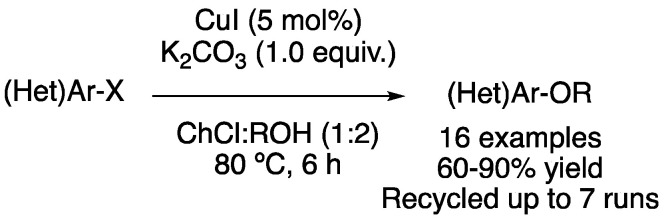
C-O cross-coupling reaction.

Cu-catalyzed Ullmann-type O-arylation has also been explored in these neoteric solvents. Specifically, mixtures of ChCl with HBD, such as ethylene glycol, glycerol, 1,3-propanediol or lactic acid, were employed as solvents and reagents to be coupled with aryl bromides or iodides, using CuI as a catalyst and K_2_CO_3_ as a base at 80 °C for 6 h. Three pharmacologically active compounds were obtained, with gram-scale synthesis being feasible. In addition, the catalyst, DES and base system could be reused for up to seven cycles without an important catalytic decrease (Figure 28) [83]. Heterogeneous C-O coupling has also been reported with Cu(I)-Benzoylthiophene-SiO_2_@Fe_3_O_4_ [84].

A table summarizing the main results for each type of reaction and conditions has been included (Table 3)

## 5. C-H Functionalization

**Figure 29:**
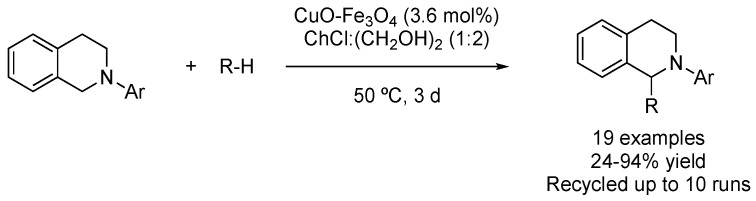
Cross-dehydrogenative coupling of 1,2,3,4-tetrahydroisoquinolines.

C-H bonds are ubiquitous in organic molecules, and direct functionalization of this functionality greatly reduces the number of steps, thereby saving solvents, reagents, energy, and time. One of the first examples of C-H functionalization in DESs was based on performing a cross-dehydrogenative coupling between 1-aryl-1,2,3,4-tetrahydroisoquinolines and different pro-nucleophiles in the mixture ChCl:(CH_2_OH)_2_ (1:2). Although a heterogeneous catalyst based on copper oxide impregnated on magnetite was used, it was reported that the active catalytic species were, probably, solubilized copper salts, with the inorganic support acting as a reservoir. The oxidant required for regenerating the catalyst was atmospheric oxygen, releasing a molecule of water as the only by-product. In addition, the eutectic mixture and catalyst could be recycled up to 10 times without losing its catalytic activity (Figure 29, Table 4, entry 1) [85].

**Figure 30:**
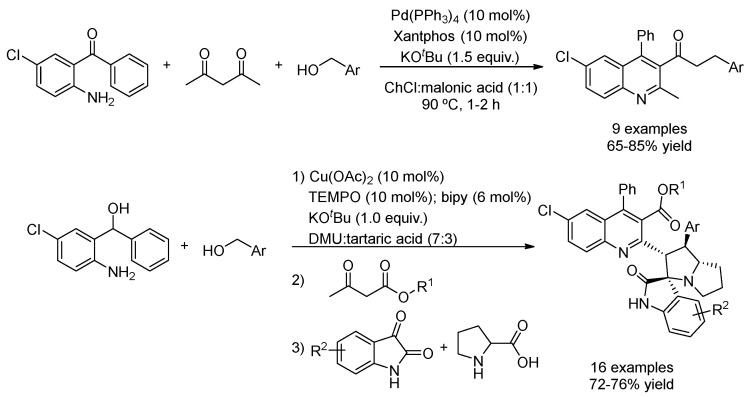
Sequential Friedländer condensation and C-H functionalization.

The acidic mixture ChCl:malonic acid (1:1) was employed as a catalytic solvent to perform the sequential Friedländer reaction followed by a Pd(PPh_3_)_4_/Xantphos-catalyzed α-alkylation of the quinolinyl methyl ketone with benzyl alcohol through a hydrogen auto-transfer reaction (Table 4, entry 2) [86]. A similar approach was also applied in the mixture DMU:tartaric acid (7:3) in the presence of an iridium catalyst to access (E)-4-benzylidenylacridines and (E)-2-styrilquinoline-3-carboxamides [87]. To further expand this strategy, the same authors also synthesized a series of spiro[indoline-3,3′-pyrrolizin]-2′-yl)-4-phenylquinoline-3-carboxylates, in this case performing the C(sp^3^)-H functionalization using Cu(OAc)_2_ and TEMPO as a catalytic system (Figure 30, Table 4, entry 3) [88].

**Figure 31:**
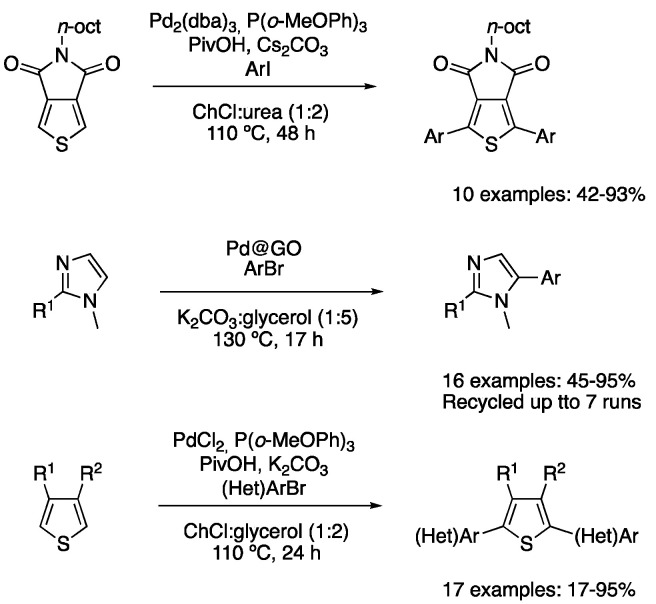
Direct arylation of heterocyclic arenes.

Regarding C-H functionalization processes, the strategy that has attracted the attention of synthetic chemists over the last decades is the C-H activation process. The first example found in the literature is the direct arylation through the C-H activation of thiophene derivatives. Pd_2_(dba)_3_ in combination with P(*o*-MeOPh)_3_ was used as a catalyst in the presence of pivalic acid and caesium carbonate for the diarylation of 5-octylthieno[3,4-*c*]pyrrole-4,6-dione with aryl iodides in ChCl:urea (1:2) as a solvent at 110 °C for 48 h. A total of 10 examples of diarylated products were reported, showing moderate-to-excellent yields for apolar aryl iodides, although a drastic decrease in the reaction yield was reported when using iodobenzene derivatives bearing nitro, acetyl or methoxy groups. However, in those cases, the addition of cyclopentyl methyl ether as a co-solvent improved the reaction yields (Table 4, entry 4) [89]. A graphene oxide-supported palladium catalyst was also employed for the direct arylation of imidazole derivatives, using K_2_CO_3_/glycerol (1:5) as a solvent. The reaction was conducted at 130 °C for 17 h using (hetero)aryl bromides as coupling partners, affording the corresponding arylated imidazoles in moderate-to-excellent yields. In addition, the catalyst could be recycled up to 10 times with only a slight decrease in the reaction yield(Table 4, entry 6) [90]. More recently, ChCl:glycerol (1:2) has been proposed as a better solvent for the arylation of thiophene derivatives. In this case, PdCl_2_ and P(*o*-MeOPh)_3_ were used as catalysts, employing pivalic acid as an additive and potassium carbonate as a base with the reaction between aryl bromides and thiophene derivatives being stirred at 110 °C for 24 h. Good-to-excellent yields were obtained, especially when electron-poor aromatic bromides were employed, yielding products with application in the photovoltaic field (Figure 31, Table 4, entry 5) [91].

A breakthrough ruthenium-catalyzed C-H activation method was recently described for the synthesis of several heteroaromatic compounds. Using ChCl:(CH_2_OH)_2_ (1:2) as a solvent, NaOAc as a base and simple [Ru(*p*-cymene)Cl_2_]_2_ as pre-catalyst, the formation of isoquinoline derivatives was achieved by the reaction between *N*-methoxybenzamide derivatives and disubstituted alkynes at 70 °C for 16 h. The same method could be efficiently applied to *N*-phenoxyacetamide, obtaining the corresponding benzofurane derivative in good yield. Electron-poor olefins could also be used, obtaining cyclic or acyclic products depending on the substrate. In addition, the reaction between benzoic acid and electron-deficient olefins was also tested, obtaining the corresponding cyclic products. In that case, the mixture betaine:HFIP (1:2) was employed as a solvent, while the catalytic system was composed of a combination of [Ru(*p*-cymene)Cl_2_]_2_ and a catalytic amount of Cu(OAc)_2_, which was employed as an oxidant. However, the final oxidant was an atmospheric oxygen. If disubstituted alkynes were used instead of olefines, the corresponding isocumarin derivatives were obtained in good-to-excellent yields. In addition, 2-thiophenecarboxylic acid could also be used as a starting material with olefines, affording the corresponding acyclic products. Finally, the same method could be applied to the reaction between 1-arylpyrazol derivatives as substrates with electron-poor olefins. Even a gram-scale reaction was reported, with the reaction being recyclable up to three times. Several green metrics were analyzed proving that the method outperforms classical approaches in terms of sustainability (Table 4, entry 7) [92].

**Figure 32:**
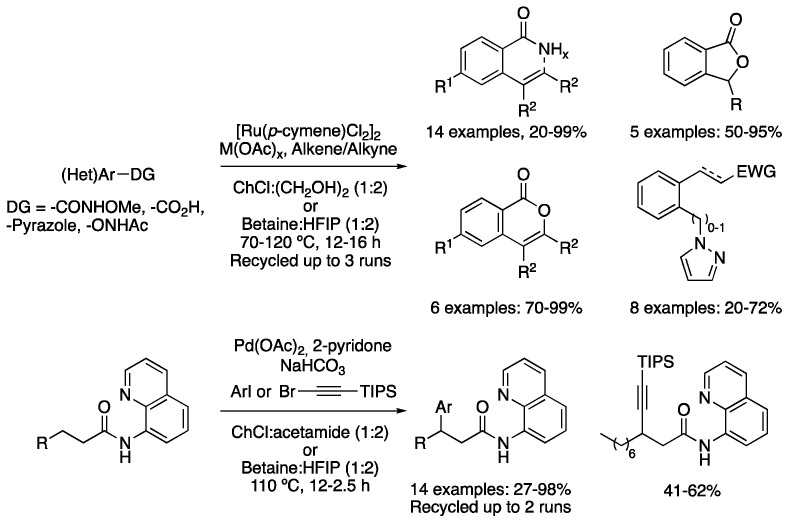
Ru and Pd catalyzed C-H functionalization.

Although the previous reports of C-H activation in DESs were focused on C*_sp_*_2_-H bond activation, a recent example describes the arylation and alkynylation of unactivated aliphatic amides through a palladium-catalyzed C*_sp_*_3_-H activation. The 8-aminoquinoline motif was used as a directing group to carry out the selective arylation at the ß-position to the carbonyl group. Pd(OAc)_2_ was used as the pre-catalyst and 2-pyridone as the ligand, as well as the inexpensive sodium bicarbonate as a base at 110 °C. Two DESs were effectively employed as solvents. On the one hand, the reaction proved to be efficiently performed in the inexpensive mixture ChCl:acetamide (1:2), obtaining moderate-to-good yields with different aliphatic amides and *p*-substituted aryl iodides in 12 h. However, the usage of betaine:HFIP (1:2) as a solvent outperformed the previous DES, with the reaction being much faster in 2.5 h. The solvent and catalyst could be recycled once, although the yield dramatically dropped after the third cycle. The directing group could be removed after the reaction in a one-pot manner by treatment with 40% H_2_SO_4_, releasing the corresponding carboxylic acid with good yield. The reaction with (bromoethynyl)triisopropylsilane was also possible under the same conditions, affording the corresponding alkynylated product without the need of using any silver salt required when the reaction is carried out in classical organic solvents. The TIPS group could then be removed and examples of traditional alkyne chemistry, such as Sonogashira coupling or CuAAC click-chemistry in DES, were reported (Figure 32, Table 4, entry 8) [93].

**Table 4:** Selected examples of C-H functionalizations catalyzed by transition metals in DESs.

Entry	Reaction	DES	Conditions	Product	Ref.
1	CDC	ChCl:(CH_2_OH)_2_ (1:2)	[Cu], 50 °C	1-ArTetrahydroisoquinolines	[85]
2	Hydrogen auto-transfer	ChCl:malonic acid (1:1)	[Pd], 90 °C	Quinolines	[86]
3	Hydrogen auto-transfer	DMU:tartaric acid (7:3)	[Cu] + TEMPO	Spiroindoles	[88]
4	Direct arylation	ChCl:urea (1:2)	[Pd], 110 °C	Thienyl derivatives	[89]
5	Direct arylation	ChCl:glycerol (1:2)	[Pd], 110 °C	Thienyl derivatives	[91]
6	Direct arylation	K_2_CO_3_:glycerol (1:5)	[Pd], 130 °C	Imidazole derivatives	[90]
7	C*_sp_*2-H activation	ChCl:(CH_2_OH)_2_ (1:2)Betaine:HFIP (1:2)	[Ru], 70–120 °C	(Het)Ar derivatives	[92]
8	C*_sp3_*-H activation	ChCl:acetamide (1:2)Betaine:HFIP (1:2)	[Pd], 110 °C	arylated amides derivatives	[93]

## 6. Multicomponent Reactions

**Figure 33:**
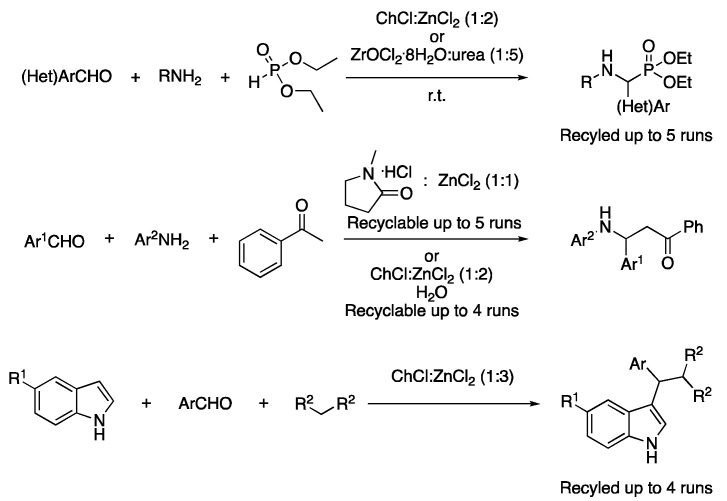
Zn-based DESs catalyzed multicomponent reactions.

A multicomponent reaction (MCR) is an organic transformation in which three or more starting materials combine to form a product containing most of the initial atoms. One of the earliest examples uses the eutectic mixture ChCl:ZnCl_2_ (1:2) as a catalyst, due to its Lewis acid character, to perform the Kabachnik–Fields reaction. A total of 15 examples were reported with yields ranging from 70 to 98%. The catalyst could be reused up to five times, showing only a slight decrease in its catalytic activity (Table 5, entry 1) [94]. Similarly, the same eutectic mixture was employed as a catalyst for performing the Mannich-type reaction of aldehyde, amines and ketones. The catalyst could be reused up to four times. In these reactions, water was the solvent, with DESs only being the catalyst of the reaction [95], as in the case of an immobilized deep eutectic solvent on a recyclable nanocomposite to perform the Mannich-type reaction where EtOH was the solvent (Table 5, entry 2) [96]. Finally, the mixture ChCl:ZnCl_2_ (1:3) was employed as a catalyst for the solvent-free condensation of indoles, aldehydes and activated methylene compounds (Figure 33, Table 3, entry 3) [97].

**Figure 34:**
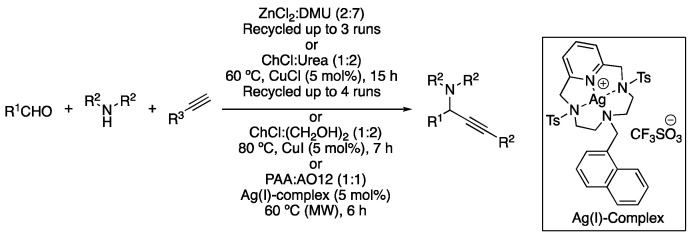
Examples of A^3^-coupling in DESs.

The use of the mixture ZnCl_2_:DMU (2:7) was reported both as a catalyst and solvent for performing the A^3^-coupling of aldehydes, amines and alkynes at 80 °C for 20 h, affording the corresponding propargyl amines. A total of 19 examples were described with yields ranging from 34–88% (Table 3, entry 4) [98]. Other examples of A^3^ coupling in DESs have been described in ChCl:urea (1:2) using CuCl as a catalyst, showing good yields at 60 °C using only 5 mol% of catalyst loading. In addition, the DES could be recycled up to four times, although a 30% loss in the catalytic activity was observed after three cycles [99]. In addition, the same authors proposed the use of salicylic aldehyde derivatives, yielding the corresponding 3-aminobenzofuran products after in situ cyclizations instead of the propargylamine. In that case, 5 mol% of CuI was employed as a catalyst and ChCl:(CH_2_OH)_2_ (1:2) as a solvent at 80 °C for 7 h (Table 4, entry 5) [100]. In addition, the A^3^ coupling in DESs has also been reported using 5 mol% of a silver complex based on a macrocyclic pyridine-containing ligand. The eutectic mixture was composed of phenylacetic acid and *N,N*-dimethyldodecylamine *N*-oxide (1:1) and the coupling reaction was performed in a microwave reactor at 60 °C for 6 h (Figure 34, Table 4, entry 6) [101].

Another type-IV DES, namely, ZrOCl_2_.8H_2_O:Urea (1:2), was used as a catalyst and solvent for the Kabachnik–Fields reaction between aldehydes, amines and dimethyl phosphite. A total of 21 examples were reported with yields ranging from 88 to 98%. These reactions were performed at room temperature and completed in 5–25 min. Once the reaction was finished, water was added, dissolving the DESs, which were then recovered and reused for up to five cycles showing only a slight loss in the catalytic activity [102].

**Figure 35:**
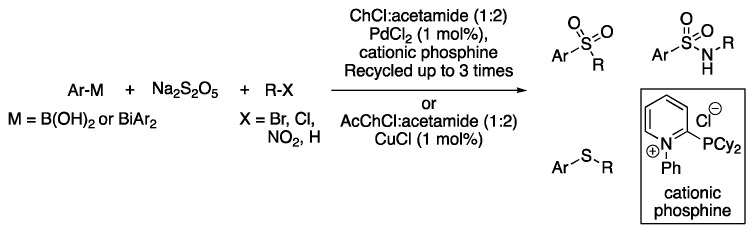
Multicomponent synthesis of sulfones, sulfides and sulfonamides.

The palladium-catalyzed synthesis of unsymmetrical substituted sulfones and sulfides is one of the first examples of multicomponent reactions in which DESs act solely as solvents. Although sulfones can be obtained by the direct insertion of SO_2_, handling this toxic gas is extremely dangerous. Therefore, the inexpensive food additive sodium metabisulfite was used as a SO_2_ surrogate. This helped to generate aryl sulfinates through the reaction of aryl boronic acids using PdCl_2_ and a cationic phosphine ligand as a catalytic system. This reaction was conducted in ChCl:acetamide (1:2) as a solvent at 80 °C in the presence of several electrophiles, affording the corresponding sulfones. The in situ generated sulfinate species could also react with I_2_ in a one-pot manner, yielding the disulfide products, which could then react with electron-rich aromatic rings or radical scavengers to afford different unsymmetrical substituted sulfides [103]. A similar strategy was developed for the synthesis of sulfonamides. Thus, using Na_2_S_2_O_5_ as a sulfur dioxide surrogate and non-toxic triarylbismuthines as aryl source sulfinates were generated. They reacted with nitrocompounds, giving an adduct that was reduced in situ by a copper-catalyzed process using NaHSO_3_ as a reductant, which comes from a decomposition product generated in the first reaction step. In this case, the mixture AcChCl:acetamide (1:2) was found to be the optimal solvent (Figure 35, Table 5, entry 7) [104].

**Figure 36:**
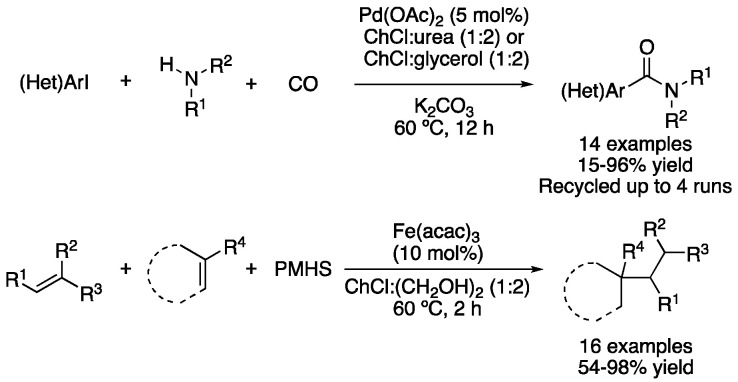
Aminocarbonylation and multicomponent radical addition.

The research group of Capriati and Salomone described another palladium-catalyzed process in the mixtures ChCl:urea (1:2) and ChCl:glycerol (1:2) as the aminocarbonylation of aryl iodides. Using palladium acetate as a catalyst, 27 atm of CO and K_2_CO_3_ as a base, primary or secondary amines and (hetero)aryl iodides were transformed into the corresponding amide products at 60 °C in 12 h. Both DESs mixtures proved to be more efficient solvents for this transformation than pure glycerol, DMF or water. Moderate-to-excellent yields were obtained with electron-deficient aryl iodides, while the presence of electro-donating groups attached to the aromatic ring produced a decrease in the reaction yield. In addition, the catalyst could be recycled up to four times with a slight decrease in the reaction yield (Table 5, entry 8) [105]. More recently, a multicomponent radical conjugate addition was described in ChCl:(CH_2_OH)_2_ (1:2). The addition of di- and tri-substituted unactivated alkenes to electron-deficient olefins was achieved by employing Fe(acac)_3_ as a catalyst and the inexpensive and non-toxic poly(methylhydrosiloxane) as a reducing agent. Reactions were carried out for 2 h at 60 °C, and 16 examples were reported with yields ranging from 48 to 98% (Figure 36, Table 5, entry 9) [106].

**Table 5:** Selected examples of MCR catalyzed by transition metals in DESs.

Entry	Reaction	DES	Conditions	Product	Ref.
1	Kabachnik–Fields	ChCl:ZnCl_2_ (1:2)ZrOCl_2_:urea (1:5)	r.t.	Aminophosphonates	[94]
2	Mannich	ChCl:ZnCl_2_ (1:2)	r.t.	Aminocarbonyls	[96]
3	Condensation	ChCl:ZnCl_2_ (1:3)	r.t.	3-indole derivatives	[97]
4	A^3^ coupling	ZnCl_2_:DMU (2:7)	r.t.	Propargylamines	[98]
5	A^3^ coupling	ChCl:urea (1:2) ChCl:(CH_2_OH)_2_ (1:2)	[Cu], 60–80 °C	Propargylamines	[99,100]
6	A^3^ coupling	PAA:AO12 (1:1)	[Ag], 60 °C	Propargylamines	[101]
7	Sulfonylation	(Ac)ChCl:acetamide (1:2)	[Pd] or [Cu], 80 °C	Sulfones/sulfonamides	[103,104]
8	Aminocarbonylation	ChCl:urea (1:2)ChCl:glycerol (1:2)	[Pd], 60 °C	(Het)Arylamides	[105]
9	Conjugate addition	ChCl:(CH_2_OH)_2_ (1:2)	[Fe]. 60 °C	Addition products	[106]

## 7. Miscellaneous

In this review, transition-metal-catalyzed reactions performed in DESs have been classified according to the nature of the described transformation for clarity. However, some reports do not fit into any of the previous reaction types and will be discussed in this section as miscellaneous reactions.

**Figure 37:**
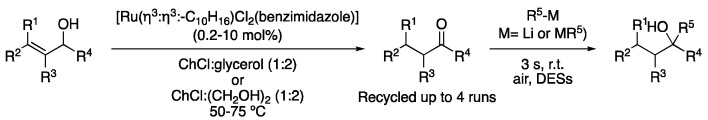
Ru-catalyzed isomerization reaction.

In 2014, the García-Álvarez group described the isomerization of allylic alcohols into carbonyl compounds in ChCl:glycerol (1:2) as a solvent. This reaction was catalyzed by the ruthenium complex [Ru(η^3^:η^3^:-C_10_H_16_)Cl_2_(benzimidazole)] in the absence of any additive at 75 °C. Excellent yields were obtained using low catalyst loadings for mono-substituted allylic alcohols in less than 20 min. However, higher catalyst loadings and reaction times were required with 1,2-disubstituted compounds and no reaction was observed in the case of using trisubstituted allylic alcohols. Only a partial deactivation of the catalyst was observed when it was reused up to four times (Table 6, entry 1) [107]. This approach was later employed in collaboration with Prof. Capriati’s group, combining the aforementioned isomerization with the addition of organometallic reagents, such as organolithium or organomagnesium species, to the in situ generated carbonylic compounds, yielding tertiary alcohols in a one-pot manner. It is worth mentioning that these reactions were performed under air atmosphere at room temperature, yielding the corresponding products in only 3 s reaction time [108]. (Figure 37). Similarly, the Pd-catalyzed cross-coupling reaction between organolithium compounds and (hetero)aryl halides was studied in DESs, obtaining good results with mixtures, such as ChCl:(CH_2_OH)_2_ (1:2). However, the best results were found using an aqueous sodium chloride solution, favoring the C-C coupling over the protonolysis [109]. The Ru-catalyzed isomerization of racemic allylic alcohols was also coupled with the enantioselective bioreduction of the in situ generated ketones to afford the corresponding enantiopure alcohols in DES-buffer mixtures [110].

Another example is the iron-catalyzed atom transfer radical polymerization of methyl methacrylate. This reaction was performed at 60 °C using several eutectic mixtures, such as acetamide:KSCN, acetamide:urea, caprolactam:urea, ChCl:urea or TBABr:glycerol, using FeBr_2_ as a catalyst, resulting in a greener and cheaper polymerization method, compared with the traditional ones [111].

**Figure 38:**
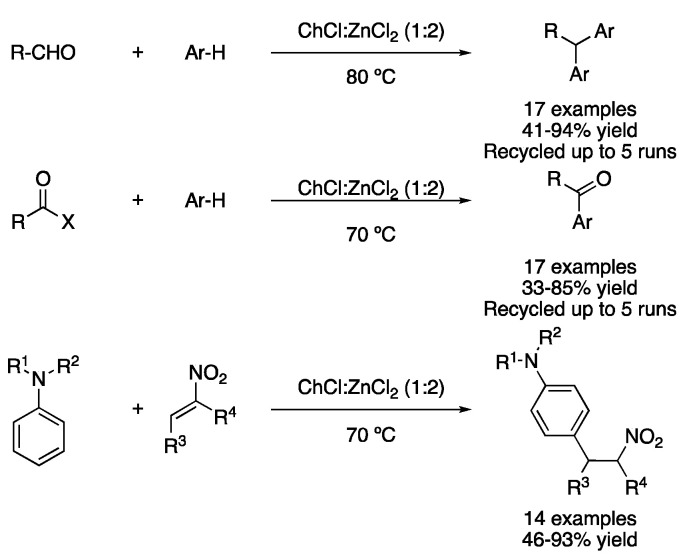
Friedel-Crafts-type alkylation/acylation.

As has been mentioned before, type I and type II DESs are formed by metal halides (hydrated or not) in combination with quaternary ammonium salts. Thus, mixtures, such as ChCl:ZnCl_2_, can be employed not only as reaction media but also as Lewis acid catalysts. Therefore, Friedel–Crafts alkylations have been widely studied in this kind of mixture. One example is the reaction between aldehydes with electron-rich arenes, affording the corresponding diarylalkanes or triarylmethanes, which are important building blocks in medicinal, materials and dye industries. In addition, DES can be dissolved in water after the reaction and then dried and reused up to five times without losing its activity (Table 6, entry 2) [112]. The same eutectic mixture was also employed to perform the acylation of electron-rich arenes using acid chlorides or anhydrides as acylating reagents; this is also recyclable up to five times (Table 6, entry 3) [113]. Finally, catalytic amounts of the ChCl:ZnCl_2_ (1:2) mixture (10 mol%) were employed in the solvent-free reaction between mono- or dialkylated anilines and β-nitrostyrenes, yielding the corresponding alkylated products in good yields and excellent regioselectivity towards the para-substituted adducts (Figure 38, Table 6, entry 4) [114].

**Figure 39:**
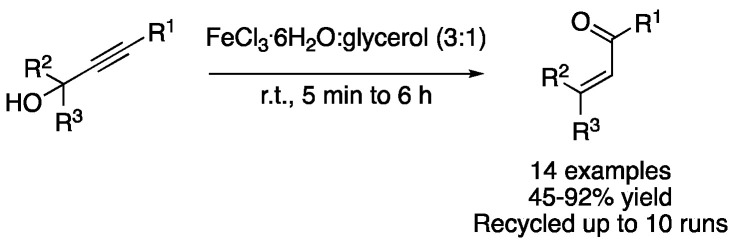
Meyer-Schuster rearrangement of propargylic alcohols.

Similarly, the iron-based eutectic mixture FeCl_3_.6H_2_O:glycerol (3:1) was described as a solvent and Lewis acid catalyst for performing the Meyer–Schuster rearrangement of propargylic alcohols to afford the corresponding ketones or aldehydes in excellent yields. Moreover, the catalytic DESs were recycled up to 10 times without losing their activity (Figure 39, Table 6, entry 5) [115].

Some examples of esterification reactions in DESs can also be found. For instance, free sugars reacted with acetic anhydride at 100 °C, affording the per-O-acetylated hemiacetals of sugars, such as d-glucose, d-galactose or d-lactose, among others. Although Brønsted acid-based type III DES, such as ChCl:oxalic acid or ChCl:malonic acid, were efficient solvents for this transformation, the best results were obtained with ChCl:ZnCl_2_ (1:2) [116]. The same strategy was also applied to the O-acetylation of chitin [117]. Similarly, the esterification of acetic and formic acids was performed in the eutectic mixture ChCl:CrCl_3_.6H_2_O (1:2). Several alcohols were used in combination with the aforementioned acids at room temperature for 24 h, obtaining moderate-to-excellent yields of the corresponding esters. In addition, the catalytic solvent could be recycled up to four times without any significant loss in its catalytic activity [118].

**Figure 40:**
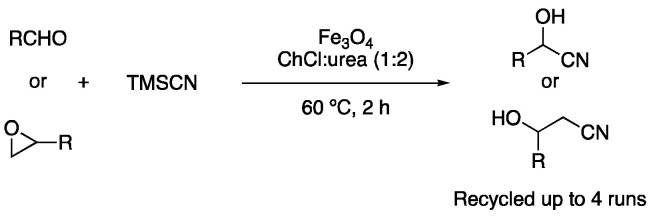
Cyanosilylation of aldehydes and epoxides.

In 2015, Prof. Azizi described the use of magnetic nanoparticles to catalyze the cyanosilylation of aldehydes and epoxides in ChCl:urea (1:2), obtaining good-to-excellent yields of the corresponding cyanohydrins in 2 h reaction time (Figure 40, Table 6, entry 6) [119].

**Figure 41:**
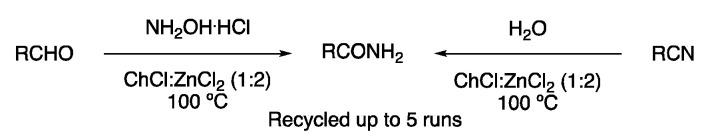
Synthesis of primary amides from nitriles and aldehydes.

To conclude, several transformations involving the synthesis of amides, ureas and carbamates in DESs will be described. One of the first reported examples was the use of ChCl:ZnCl_2_ (1:2) as a catalyst and solvent to carry out the synthesis of primary amides from aldehydes and nitriles. When aldehydes were used as starting materials, they reacted with hydroxylamine hydrochloride in ChCl:ZnCl_2_ (1:2) at 100 °C, while in the case of nitriles, a mixture of DESs (4 g) and water (1 g) was employed as a solvent to afford the corresponding amides. The catalyst could be recycled up to five times, showing a higher decrease in the catalytic activity after the third cycle in the case of the synthesis through aldehydes [120]. The formylation of aniline derivatives was also studied under solvent-free conditions using a mesoporous heterogeneous catalyst functionalized with the eutectic mixture N-methylpyrrolidonium:ZnCl_2_ (Figure 41, Table 6, entry 7) [121].

**Figure 42:**
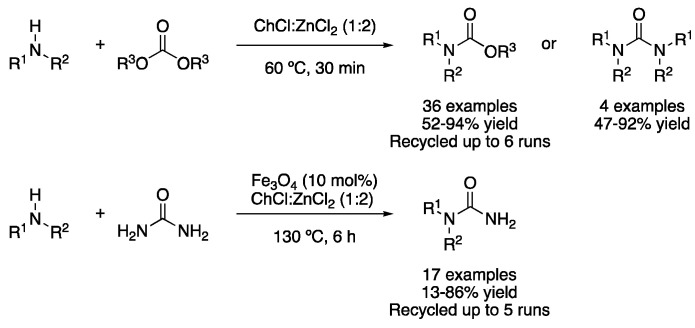
Synthesis of carbamates and urea derivatives.

The synthesis of carbamates was also performed using the mixture ChCl:ZnCl_2_ (1:2). Thus, a series of primary and secondary amines were reacted with two equivalents of carbonates to yield the corresponding carbamate derivatives at 60 °C in just 30 min. In addition, some examples of urea derivatives were reported under the same reaction conditions by changing the stoichiometry of the starting materials. The eutectic mixture could be recycled up to six times without any loss of catalytic activity [122]. Other carbamates and monosubstituted ureas were prepared in a similar fashion using a heterogeneous catalyst based on superparamagnetic Fe_3_O_4_ nanoparticles in the mixture ChCl:ZnCl_2_ (1:2) (Figure 42, Table 6, entry 8) [123]. Finally, N-acylureas were prepared using N,N′-dicyclohexylcarbodiimide (DCC) and benzoic acid derivatives at 60 °C in the mixture ChCl:urea (1:2) as a solvent. This reaction was catalyzed by copper oxide supported in magnetic iron nanoparticles [124].

**Table 6:** Selected examples of miscellaneous reactions catalyzed by transition metals in DESs.

Entry	Reaction	DES	Conditions	Product	Ref.
1	Allylic alcohol isomerization	ChCl:glycerol (1:2)	[Ru], 50–75 °C	ketones	[107,108]
2	Friedel–Crafts alkylation	ChCl:ZnCl_2_ (1:2)	80 °C	Ar-R	[112]
3	Friedel–Crafts acylation	ChCl:ZnCl_2_ (1:2)	70 °C	ArCOR	[113]
4	Friedel–Crafts alkylation	ChCl:ZnCl_2_ (1:2)	70 °C	Alkylated anilines	[114]
5	Meyer–Schuster	FeCl_3_.6H_2_O:glycerol (3:1)	r.t.	Ketones/aldehydes	[115]
6	Cyanosilylation	ChCl:urea (1:2)	[Fe], r.t.	cyanohydrins	[119]
7	Amide synthesis	ChCl:ZnCl_2_ (1:2)	100 °C	Primary amides	[121]
8	Substitution	ChCl:ZnCl_2_ (1:2)	[Fe], 60–130 °C	Carbamates/ureas	[122,123,124]

## 8. Conclusions

Deep eutectic solvents were proposed more than a decade ago as an alternative reaction media in organic synthesis. Although their physicochemical properties seemed to differ too much from traditional volatile organic solvents and, therefore, several limitations were found at the beginning; nowadays, DESs have clearly become a serious option to take into consideration when planning a synthetic method. Types I, II and IV DESs, which contain a metal salt in their structure, can be used as recyclable solvents and catalysts for several organic transformations. Meanwhile, the knowledge about the compatibility of homogeneous and heterogeneous metallic catalysts in types III and V DESs has greatly improved over the last few years. This has allowed the catalyst/DES system design to improve the achieved selectivities and efficiencies. As a result, eutectic mixtures are no longer trivial solvents for very specific reactions and have proven to be a useful alternative to traditional solvents in transition-metal-catalyzed organic transformations. In most cases, the possible recovery and recycling of the catalyst/DES system have greatly enhanced the efficiencies of the reactions. The use of DESs has not only increased the sustainability of the reaction due to the benign properties of the solvents but has also allowed the use of milder conditions, in terms of the applied temperature and pressures, to perform some transformations; this significantly reduces the energetic costs. In addition, DESs have proven to not only be alternative solvents for known reactions but also to offer novel and exciting reactivities due to their unique properties, as has been shown, for instance, in the sulfonylation reaction. For these cases, the right choice of DES and the correct design of the metal–ligand are crucial to successfully obtaining the expected product. There is a clear trend towards increasing the popularity of DESs and the applications and knowledge about these solvents will keep growing in the coming years.

## Figures and Tables

**Figure 3:**
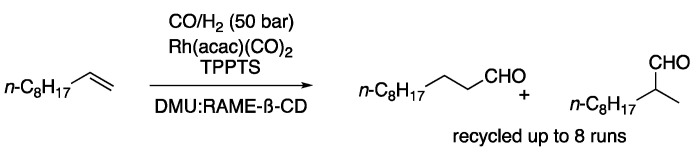
Hydroformylation of 1-decene.

**Figure 25:**
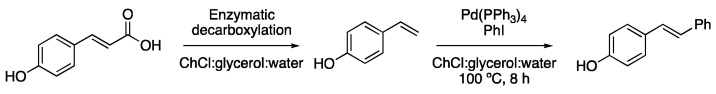
One-pot decarboxylation and Heck cross-coupling.

**Table 3:** Selected examples of cross-coupling reactions catalyzed by transition metals in DESs.

Entry	Reaction	DES	Conditions	Product	Ref.
1	Stille	Sugar:DMU:NH_4_Cl	[Pd], 90 °C	Biaryl	[45]
2	Suzuki	Sugar:DMU:NH_4_Cl	[Pd], 90 °C	Biaryl	[46]
3	Suzuki	ChCl:glycerol (1:2)	[Pd], 60–100 °C	(Het)biaryl	[48,49,50,56,61]
4	Suzuki	ChCl:(CH_2_OH)_2_ (1:2)	[Pd], 100 °C	Biaryl	[53,54]
5	Suzuki	ChCl:glycerol (1:2)	[Pd], 90 °C	Imidazole-fused deriv.	[52]
6	Suzuki	ChCl:urea (1:2)	[Ni], 60 °C	(Het)biaryl	[57]
7	Heck	l-carnitine:urea	[Pd], 80 °C	Ar-CH=CHR	[58]
8	Heck	ChCl:glycerol (1:2)	[Pd], 80–120 °C	Ar-CH=CHR	[48,62]
9	Heck	ChCl:(CH_2_OH)_2_ (1:2)	[Pd], 120 °C	Ar-CH=CHR	[53]
10	Heck	AcChCl:urea (1:2)	[Pd],120 °C	Ar-CH=CHR	[54]
11	Sonogashira	d-mannose:DMU (7:3)	[Pd], 80 °C	Ar-C≡C-Ar’	[58]
12	Sonogashira	Ph_3_PMeBr:glycerol (1:2)	[Pd], 60 °C	Ar-C≡C-Ar’	[48,53]
13	Sonogashira	AcChCl:urea (1:2)	[Pd], 60 °C	Ar-C≡C-Ar’	[54]
14	Hiyama	ChCl:glycerol (1:2)	[Pd], 100 °C	Ar-R	[53,54,74]
15	Tsuji–Trost	RAME-ß-CD:urea (3:7)	[Pd], 90 °C	Allylamine	[10]
16	Negishi	ChCl:urea (1:2)	[Pd], 60 °C	Ar-R	[75]
17	Ullmann C-N	ChCl:glycerol (1:2)	[Cu], 60–100 °C	ArNR^1^R	[76,78]
18	Goldberg	ChCl:H_2_O (1:2)	[Cu], 80 °C	Amides	[77]
19	Ullmann C-O	ChCl:ROH	[Cu], 80 °C	Ar-OR	[83]

## Data Availability

Not applicable.

## Abbreviation

acac	acetylacetonate
AcChCl	Acetylcholine chloride
API	Active Pharmaceutical Ingredient
β-CD	β-cyclodextrin
ChCl	Choline chloride
CPME	Cyclopentyl ethyl ether
CuAAC	Cu(I)-catalyzed azide–alkyne cycloaddition
DABCO	1,4-Diazabicyclo[2.2.2]octane
dba	Dibenzylideneacetone
DCC	Dicyclohexylcarbodiimide
DES	Deep Eutectic Solvent
DFT	Density Functional Theory
DMF	Dimethyl formamide
DMU	1,3-Dimethylurea
dppf	1,1′-Ferrocenediyl-bis(diphenylphosphine)
GO	Graphene Oxide
HBA	Hydrogen bond acceptor
HBD	Hydrogen bond donor
HFIP	1,1,1,3,3,3-Hexafluoro-2-propanol
HMF	5-Hydroxymethyl furfural
MCR	Multicomponent Reaction
MW	microwave
NADES	Natural Deep Eutectic Solvent
NP	Nanoparticle
PEG	Polyethylene glycol
PivOH	Pivalic acid
TBAB	Tetrabuthyl ammonium bromide
TEM	Transition Electron Microscopy
TEMPO	2,2,6,6-Tetramethylpiperidine 1-oxyl
TIPS	triisopropylsilane
TPPTS	Triphenylphosphine-3,3′,3′′-trisulfonic acid trisodium salt
VOC	Volatile Organic Compound
XPS	X-ray photoelectron spectroscopy
